# Low-Density Geopolymer Composites for the Construction Industry

**DOI:** 10.3390/polym14020304

**Published:** 2022-01-13

**Authors:** Van Vu Nguyen, Van Su Le, Petr Louda, Michał Marek Szczypiński, Roberto Ercoli, Vojtěch Růžek, Piotr Łoś, Karol Prałat, Przemysław Plaskota, Tadeusz Pacyniak, Katarzyna Ewa Buczkowska

**Affiliations:** 1 Department of Material Science, Faculty of Mechanical Engineering, Technical University of Liberec, Studentska 2, 461 17 Liberec, Czech Republic; longsuvp90@gmail.com (V.S.L.); petr.louda@tul.cz (P.L.); michal.szczypinski@tul.cz (M.M.S.); vojtech.ruzek@tul.cz (V.R.); piotr.los@tul.cz (P.Ł.); katarzyna.ewa.buczkowska@tul.cz (K.E.B.); 2 Department of Pure and Applied Sciences, University of Urbino Carlo Bo, Via Cà Le Suore, 2/4, 61029 Urbino, Italy; r.ercoli@campus.uniurb.it; 3 Faculty of Civil Engineering, Mechanics and Petrochemistry, Warsaw University of Technology, Łukasiewicza 17, 09-400 Plock, Poland; karol.pralat@pw.edu.pl; 4 Department of Acoustics, Multimedia and Signal Processing, Wroclaw University of Science and Technology, Wybrzeże Stanisława Wyspiańskiego 27, 50-370 Wrocław, Poland; przemyslaw.plaskota@pwr.edu.pl; 5 Department of Materials Technology and Production Systems, Faculty of Mechanical Engineering, Lodz University of Technology, Stefanowskiego 1/15, 90-001 Lodz, Poland; tadeusz.pacyniak@p.lodz.pl

**Keywords:** light-weight geopolymers, polystyrene, ceramic microsphere, Liapor, carbon fiber grid, walling materials

## Abstract

The article presents preliminary results in studying reinforced and light-weight geopolymers, which can be employed in buildings, especially for walling. Such materials are very promising for the construction industry having great potential due to their favorable properties such as high mechanical strengths, low thermal conductivity, and low density. Moreover, they also exhibit several advantages from an economic and ecological point of view. The present study exanimated the use of specific fillers for the metakaolin-based light-weight geopolymers, emphasizing the above-mentioned physical properties. This research also investigated the electromagnetic shielding ability of the carbon grid built into the light-weight geopolymer structure. According to the study, the most suitable materials to be used as fillers are polystyrenes, along with hollow ceramic microsphere and Liapor. The polystyrene geopolymer (GPP) achieves five times lower thermal conductivity compared to cement concretes, which means five times lower heat loss by conduction. Furthermore, GPP is 28% lighter than the standard geopolymer composite. Although the achieved flexural strength of GPP is high enough, the compressive strength of GPP is only 12 MPa. This can be seen as a compromise of using polystyrene as a filler. At the same time, the results indicate that Liapor and hollow ceramic microsphere are also suitable fillers. They led to better mechanical strengths of geopolymer composites but also heavier and higher thermal conductivity compared to GPP. The results further show that the carbon grid not only enhances the mechanical performances of the geopolymer composites but also reduces the electromagnetic field. Carbon grids with grid sizes of 10 mm × 15 mm and 21 mm × 21 mm can reduce around 60% of the Wi-Fi emissions when 2 m away from the signal transmitter. Moreover, the Wi-Fi emission was blocked when the signal transmitter was at a distance of 6 m.

## 1. Introduction

Portland cement is currently the most important building material in the world. Despite the obvious advantages, Portland cement needs to be replaced with more environmentally friendly building materials; b its manufacture causes 8% of the global carbon dioxide emissions [1]. Furthermore, geopolymer composites perform better than conventional cement-based composites [2,3,4]. The quest for substantial construction resources is becoming ever more urgent, and geopolymer composites show promising alternative green solutions.

Geopolymers are suitable as building materials in many ways, including favorable mechanical and physical properties, high compressive strength, low thermal conductivity, etc. Light-weight geopolymers are light-weight and have even better thermal conductivity and sound insulation [5]. These excellent properties are useful as both wall and wall cladding materials as they are able to minimize heat loss and improve sound insulation in buildings. However, little attention is paid to the subject in the literature. The main objective of the current work is to investigate several potential light-weight geopolymer composites for wall and wall cladding materials. In addition, a material that can absorb electromagnetic signals is desirable. Electromagnetic waves such as cell phone signals, Wi-Fi, etc., are indispensable for modern life. The benefits of these electromagnetic signals are undeniable. However, there are hundreds of studies showing the relationship between electromagnetic radiation and health risks: being continuously exposed to these electromagnetic (EM) radiations, people can face several health risks. Wi-Fi signal (2.4 GHz) has been shown to significantly damage sperm count [6]. Male sperm counts can decrease by over 50% [7], accompanied by sperm quality in all advanced technology countries. ER radiation has negative effects on mental health and sleeping quality [8,9,10]. In addition, electromagnetic radiation impairs the functioning of electronic devices and damages electronic storage devices [11]. EM radiation could lead to information leaks or hacking [12,13]. Metals are reasonable solutions for shielding strong electromagnetic radiation because of their high electrical conductivity. However, the use of metals for EM shielding requires high installation and maintenance costs [14] and can be challenging to be employed for construction.

The current work investigated the use of light-weight geopolymers as alternative building materials. The following work evaluated the mechanical strengths, the thermal conductivity, and the density of the geopolymer composites studied. In addition, the electromagnetic shielding ability of geopolymers reinforced by the carbon grid was evaluated.

## 2. Materials and Methods

### 2.1. Materials

The raw materials used in the study, which were mixed to synthesize the light-weight geopolymers, are described as follows. 

The commercial geopolymer “Baucis k” (Ceske Lupkove Zavody, city, Czech Republic) is composed of metakaolin (Mefisto L05) and potassium alkaline activator [15].

**Figure 1:**
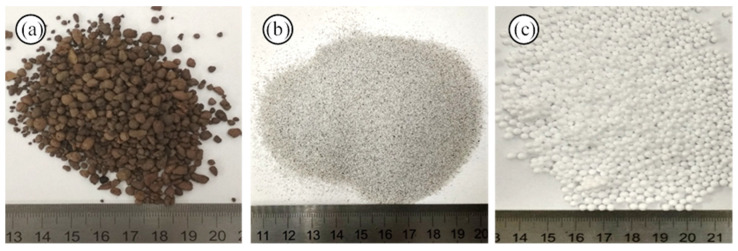
Raw materials used as fillers: (**a**) Liapor, (**b**) glass microspheres, and (**c**) EPS polystyrene.

Liapor (DEK, Vintířov, Czech Republic) (Figure 1a) is the trade name of a light-weight ceramic aggregate made from plasticity clays. It has an almost spherical shape with an average particle size ranging between 1 and 4 mm. Liapor is chemically stable and is a great alternative aggregate because of its low density (0.5 g/cm^3^) and thermal conductivity (0.11 W/m·K).

Other low thermal conductivity aggregates (filler) suitable for constructions are hollow ceramic microsphere (Figure 1b) and polystyrene (Figure 1c). Polystyrene is a synthetic polymer made from the styrene monomer. The polystyrene used in this study is expanded polystyrene (Synthos, city, Poland); it has a mean range particle size of 1–1.8 mm, a density of 0.18 g/cm^3^, and a thermal conductivity of 0.08 W/m·K. Instead, the hollow ceramic microsphere (Elite Industrial, manufacturer, city, China) has a range particle size of 0.037 to 0.425 mm, a density of 0.35 to 0.45 g/cm^3^, and a thermal conductivity of 0.11 W/m·K. 

Silica fume is a by-product of the production of elemental silicon or alloys containing silicon in electric arc furnaces [16]. Silica fume significantly improves the overall compressive strength and bond strength of the geopolymer composites [4,17,18]. Basalt fiber help improve the mechanical properties and fire resistance of the building materials [19,20]. These two materials were used to improve the overall properties of all samples studied.

### 2.2. Sample Preparation

The raw materials were mixed with weight ratios as reported in Table 1. The parametric study on the filler proportion was also performed out to better understand its influence on the performance of the sample. First, metakaolin powder (Mefisto L05) was mechanically stirred in the KOH-aqueous alkaline solution for four minutes at high speeds by means of the Eibenstock Automix 1801. The ground basalt fibers, and micro-silica (silica fume) were then added to the geopolymer mortar, and the mixing process was continued until the blend was homogenized. Finally, either one of these fillers (microspheres, Liapor, and polystyrene) were added to the geopolymer mortar to create the different geopolymers (GPs). The final geomortar was cast into the molds and tightly covered with plastic wrappers. The samples were cured for 28 days at room temperature.

### 2.3. Mechanical Strength Tests

The mechanical strengths of the geopolymers were assessed with the Instron model 4202 testing machine using the ČSN EN 1015-11 standard [21]; the reader is referred to [22] for further details. The three-point bending tests were used to evaluate the flexural strengths. Specimens with dimensions of 30 × 30 × 150 mm and 40 × 40 × 40 mm were used for flexural and compressive strength tests, respectively. The compression and bending tests were conducted with a load cell of 10 kN at a crosshead speed of 2.0 mm, at a laboratory temperature of 22 ± 3 °C. Average values of the compressive and flexural strengths were determined from the measurements of three samples in each series. The compressive strength (*f*-MPa) was calculated by:

(1) $$f=\frac{F_{max}}{A_{c}}$$

where $F_{max}$ (N) is the maximum applied load recorded by the test, and $A_{c}$ is the cross-section area of the sample [mm^2^]. 

The flexural strength (*R*-MPa) was calculated by:

(2) $$R=\frac{3\;F_{max}\;L}{2\;b\;h^{2}}$$

where L is the span length [mm], *b* [mm] is the width, and *h* [mm] is the specimen thickness.

### 2.4. Physical Properties Measurements

**Figure 2:**
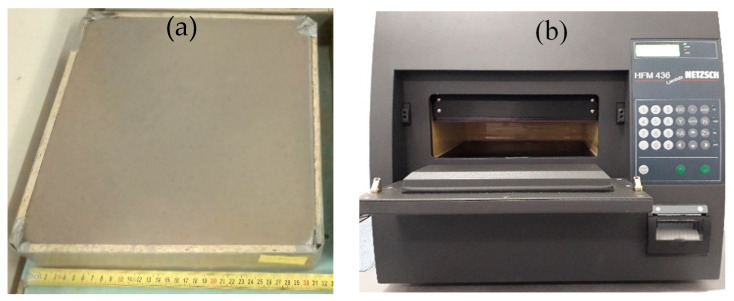
Sample GP (**a**), and thermal conductivity device: HFM436 Lambda (**b**).

The average density and thermal conductivity are the main properties for which the low-density geopolymer composites (GP) were analyzed. The GP samples with the dimensions of 300 × 300 × 50 mm were used to measure the physical properties (Figure 2a). Four specimens from each group were tested, and their mean values were calculated for the physical properties of GP. The thermal conductivity was measured with the instrument model HFM436 Lambda (Netzsch, a.s., Selb, Germany) (Figure 2b). The density of GPs was measured according to the ČSN EN 1936 [23] and estimated by dividing the mass of the sample by its volume.

### 2.5. Electromagnetic Shielding Measurements

**Figure 3:**
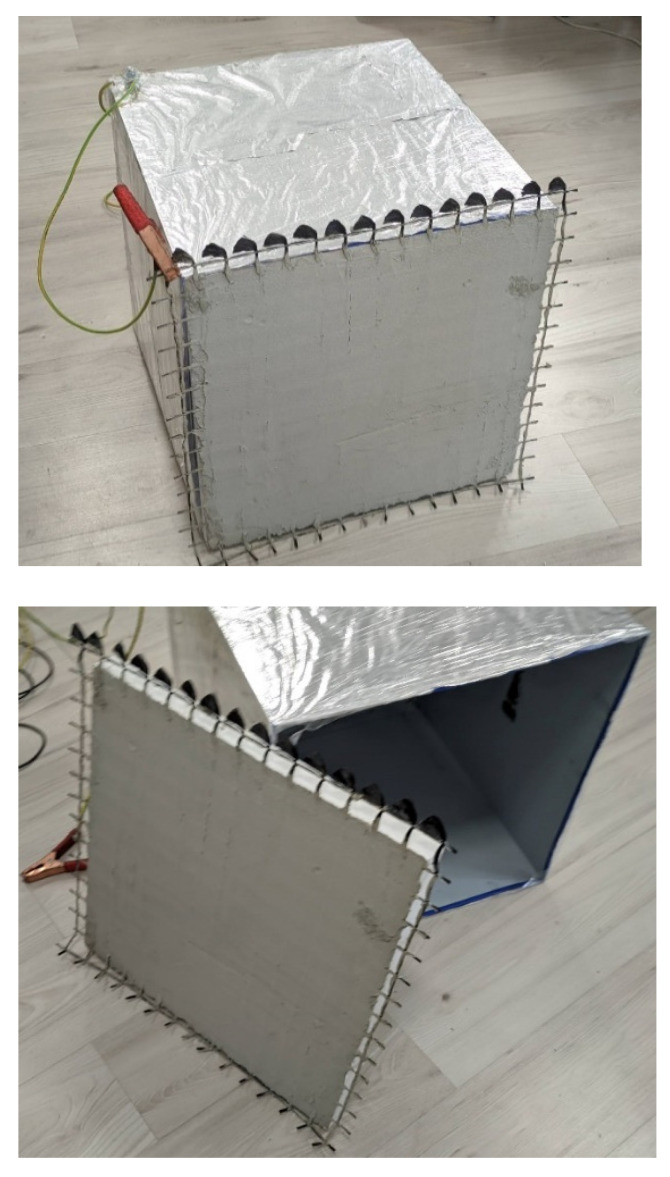
The box used for electromagnetic tests.

As a quick assessment, the experimental setup aims to measure the signal strength between the transmitter and the receiver at different distances. The electromagnetic shielding experiments were conducted using the shielded box method. The transmitter (signal source) was placed in a cubic steel box of 500 × 500 × 500 mm. Five out of six faces of the box were mounted with metal plates of 0.45 mm thick; then they were covered with aluminum foil with a thickness of 20 µm. This setup eliminates the signal transmission from the transmitter to the environment through these five faces. The geopolymer sample with the carbon grid built-in was fixed to the remaining face (see Figure 3).

For the tests, three commercially available carbon net types (Hitexbau, Augsburg, Germany) were used, which differ in their grid openings: HTC 10/15–40, HTC 21/21–40, HTC 34/34–40. Their properties are shown in Table 2.

A Qualcomm MDM9207 chipset with a Wi-Fi module was used as the signal generator. A device equipped with the MediaTek MT8735 chipset was used as the receiver, and the signal strengths were measured with the Wi-Fi Analyzer software. Both devices were tuned to a frequency of 2432 MHz. Measurements of the signal strength were performed between the transmitter and the receiver at specific distances.

## 3. Results

### 3.1. Mechanical Properties

Mechanical properties are the most relevant factors to evaluate geopolymer performance. 

**Figure 4:**
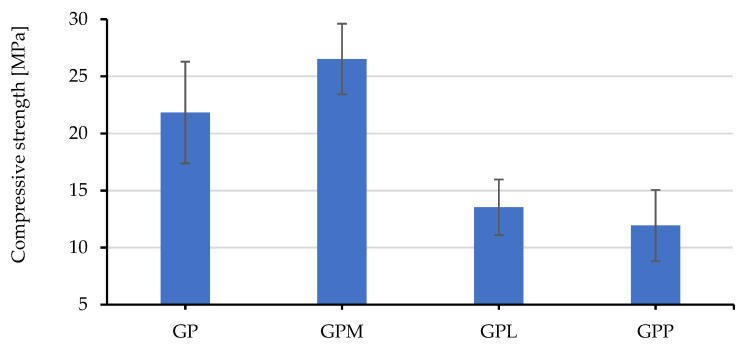
The compressive strengths of the studied samples.

Figure 4 show the compressive strengths of each sample compared to one of the porous geopolymer composite (GP-the reference). As can be observed, only GPM showed a real improvement over the reference. The microsphere is a great combination as it increased the compressive strength of the light-weight geopolymers. In contrast, the addition of Liapor and polystyrene decreased the geopolymer performance. Liapor is a light-weight expanded clay aggregate that is often used as a building material due to its hardness. However, mechanical bonding connection between Liapor and the binder did not appear to be well established, which is mainly due to its smooth surface.

The compression performance of the GPP was the worst compared to the others. Polystyrene is very light-weight and soft. Understandably, this filler lowered the compressive strength of the geopolymer composites.

**Figure 5:**
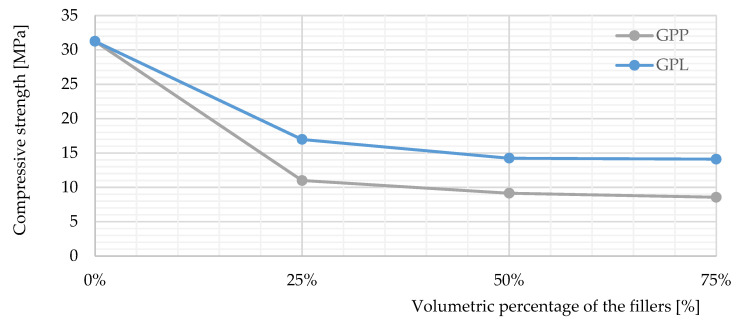
The compressive strength profiles of GPL and GPP through the volume fraction of the fillers.

To better understand the performance of the two fillers in geopolymer composites, their volume fractions were studied in samples ranging from 0 to 75 V%. The results are shown in Figure 5. In general, the compressive strengths decreased sharply when the fillers were increased to 30 volume percent. A further increase in the amount of filler does not significantly affect the compression of the GPP and GPL specimens. In return, these geopolymers achieved a low weight as well as a low thermal conductivity and a high level of sound insulation.

**Figure 6:**
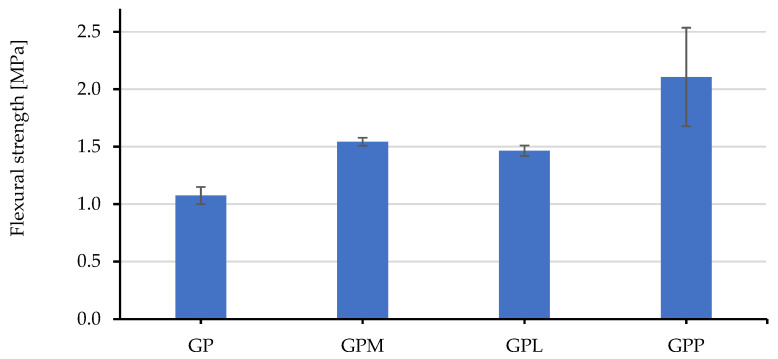
The flexural strengths of the samples compared to GP.

Figure 6 shows the flexural strengths of the geopolymers examined. In contrast, GPP performed the best on compressive strength, which was twice that of the reference. It may be due to the elasticity of the polystyrene, whereas the light-weight geopolymer was less flexible due to the high porosity. GPM and GPL also outperformed the geopolymer by around 50%. By using the fillers, the samples significantly improved the flexural strength of the material.

**Figure 7:**
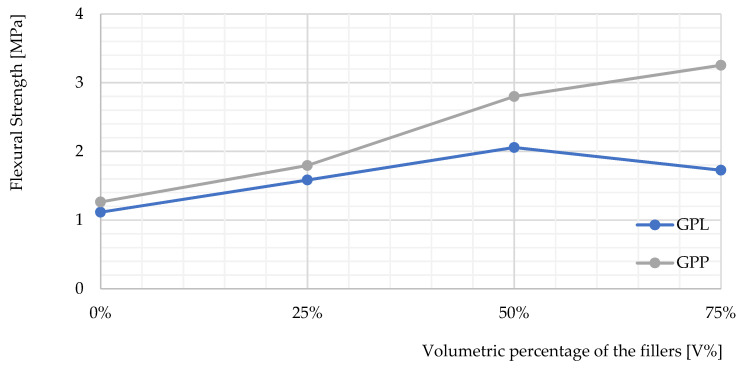
The flexural strengths profiles of GPL and GPP on the relative fillers expressed as a volumetric percentage.

Figure 7 shows the performance of the material as the filler amount increases. The flexural strengths of the samples increased as the volumetric percentage of the fillers increased. Interestingly, the flexural strength of the GPP increased over the entire test and reached three times that of the reference sample with a polystyrene content of 75% by volume. Certainly, the high flexibility of the polystyrene spheres contributes to the great flexibility performance of GPP. It should be noted that the contact areas at the three points on the test device are small. Thus, this could be another reason for GPP’s high performance. With a higher proportion of polystyrene, GPP was able to withstand a significantly higher impact strength compared to the other samples. The bending performance of GPL decreased noticeably as the proportion of Liapor exceeded 50% of the volume percentage. 

### 3.2. Thermal Conductivity and Density

**Figure 8:**
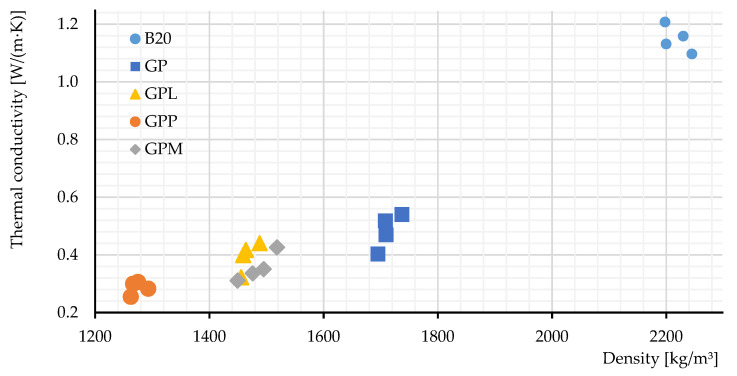
Physical properties of the samples studied.

Figure 8 show the thermal conductivities and densities of the materials examined. Portland cement concrete is used as a reference because it is the most widely used material in the construction industry [1,24]. Portland concrete (rank M250-B20) was used as a reference for this comparison.

The results show the improvement of the investigated geopolymers in terms of thermal conductivity and density. The thermal conductivity of GP is around 0.45 W/m·K, which is ~2.5 times lower than that of M250. Still, GPL and GPM achieved even better results, with an average of 0.4 and 0.3 W/m·K, respectively. In this regard, GPP achieved the best results with only ~0.25 W/m·K, i.e., 2× lower than that GP and 5× lower than M250. It is easy to understand that the heat loss caused through conduction could be reduced by two to five times.

The low density is another advantage of the light-weight geopolymers compared to Portland cement. Their density (GP) is ~1700 kg/m^3^, 27% lower than M250. GPL and GPM are even lighter, and their densities are ~1450 kg/m^3^. Finally, the best performance in terms of density is GPP, with an average density of 1250 kg/m^3^. GPP is almost two times lighter than Portland cement. This property alone makes GPP an extremely competitive alternative in the construction industry, especially as walling material.

### 3.3. Electromagnetic Shielding Properties of Carbon Nets

**Figure 9:**
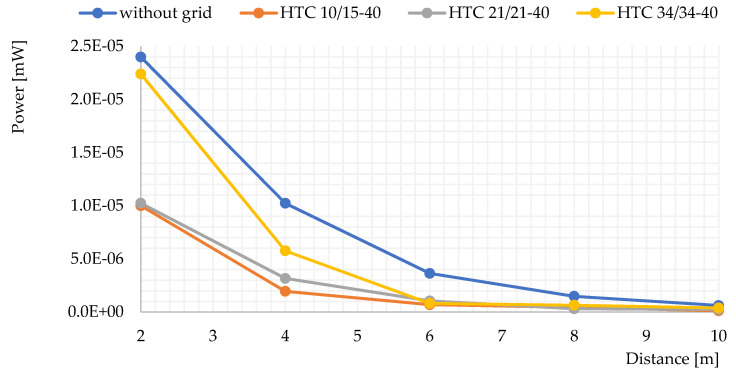
Signal shielding performance of the carbon grids along the test area.

Four series of tests were taken to evaluate the electromagnetic shielding. The standard geopolymer (GP) was used in this assessment. The measurements were carried out at a distance of 2 to 10 m from the signal source (see Figure 9). Generally, the carbon grids significantly decrease the signal. The signal was completely blocked when the distance reached 6m. The performance of HTC 10/15 and 21/21 are similar in the whole tested range. They blocked around 60% of the Wi-Fi signal at a distance of 2 m and up to 100% of the signal at a distance of 6 m. HTC 34/34 performed significantly less favorably: it only blocked 5% of the signal power at a distance of 2 m. The grid opening is a key factor not only for enhancing the mechanical properties [20], but also the electromagnetic shielding capability of the geopolymer composites.

According to the results, HFC 21/21 is the most suitable for the current purpose. However, the signal shielding performance of the carbon grid was not as good as expected. The electromagnetic signal is still quite significant up to the distance of 4 m. Chopped carbon fiber could be used to further improve the shielding performance.

## 4. Conclusions

The paper presented the study using light-weight geopolymers as alternatives for the construction industry. The research aims to find a balance between the mechanical properties and the thermal conductivity to ensure the usability of the composite materials.

The study proved that polystyrene, Liapor, and hollow ceramic microsphere are suitable fillers for light-weight geopolymers that can be used as walling materials in buildings. GPP is the most suitable walling material because its thermal conductivity is five times lower compared to Portland cement. In addition, the density of GPP is about 80% lower than that of M250. GPP significantly reduced the compressive strength as the polystyrene content increased up to 30% by volume. This fact can be seen as a disadvantage of GPP. However, this compressive strength is high enough for several applications in the construction industry. The results indicated the great potential of these materials for the building industry. The fillers are reasonable solutions to improve the required properties as discussed above and to reduce material costs.

The use of carbon grids effectively suppressed electromagnetic emissions. HTC 10/15–40 and HTC 21/21–40 yielded the best results. Certainly, the grid opening size influences the shielding performance. The work showed the necessity of further improvements for the electromagnetic shielding capability of the geopolymer composites. This can be achieved by using chopped carbon fibers as a filler for the geopolymers. The behavior of chopper carbon fibers compared to the other fillers, such as polystyrene, will be carefully evaluated in future works.

## Figures and Tables

**Table 1:** Mixing proportions of the geopolymer samples.

BGF Sample	Binder		Silica Fume [%]	Basalt Fibers [%]	Fillers [Volume %]	Filler Types
	Metakaolin [%]	Alkaline Activator [%]				
GP	100	90	10	1	-	-
GPM					30	Microspheres
GPL					30	Liapor
GPP					30	Polystyrene

**Table 2:** Physical characteristics of the carbon nets.

Carbon Net Type	Crossways [mm]	Lengthways [mm]	Density [g/cm^3^]	Tensile Strength Lengthways [N/mm^2^]	Tensile Strength Crossways [N/mm^2^]
HTC 10/15–40	10	15	1.8	2551	2847
HTC 21/21–40	21	21	1.8	2531	2841
HTC 34/34–40	34	34	1.8	2544	2720

## Data Availability

Not applicable.
